# The *Lotus japonicus* Ubiquitin Ligase SIE3 Interacts With the Transcription Factor SIP1 and Forms a Homodimer

**DOI:** 10.3389/fpls.2020.00795

**Published:** 2020-06-12

**Authors:** Yong Feng, Ping Wu, Weiwei Fu, Liwei Peng, Hui Zhu, Yangrong Cao, Xinan Zhou, Zonglie Hong, Zhongming Zhang, Songli Yuan

**Affiliations:** ^1^State Key Laboratory of Agricultural Microbiology, Huazhong Agricultural University, Wuhan, China; ^2^Key Laboratory of Biology and Genetic Improvement of Oil Crops, Ministry of Agriculture and Rural Affairs of People’s Republic of China, Oil Crops Research Institute of Chinese Academy of Agriculture Sciences, Wuhan, China; ^3^Department of Plant, Soil, and Entomological Sciences and Program of Microbiology, Molecular Biology and Biochemistry, University of Idaho, Moscow, ID, United States

**Keywords:** rhizobium-legume symbiosis, symRK, SIE3, SIP1, protein dimerization, symbiosis signaling

## Abstract

The symbiosis receptor kinase SymRK plays an essential role in symbiotic signal transduction and nodule organogenesis. Several proteins bind to SymRK, but how the symbiosis signals are transduced from SymRK to downstream components remains elusive. We previously demonstrated that both SymRK interacting protein 1 (SIP1, an ARID-type DNA-binding protein) and SymRK interacting E3 ligase [SIE3, a RING (Really Interesting New Gene)-containing E3 ligase] interact with SymRK to regulate downstream cellular responses in *Lotus japonicus* during the legume-rhizobia symbiosis. Here, we show that SIE3 interacts with SIP1 in both yeast cells and *Nicotiana benthamiana*. SIE3 associated with itself and formed a homodimer. The cysteine 266 residue was found to be essential for SIE3 dimerization and for promoting nodulation in transgenic hairy roots of *L. japonicus*. Our findings provide a foundation for further investigating the regulatory mechanisms of the SymRK-mediated signaling pathway, as well as the biological function of E3 ligase dimerization in nodule organogenesis.

## Introduction

The establishment of the rhizobia-legume symbiosis is a tightly regulated process that integrates bacterial infection steps with specialized organ development. Nodule formation is activated in response to rhizobia-derived nodulation factors (NFs), a group of lipochitooligosaccharides (LCOs) with different host-specific decorations (Long, 1989; Lerouge et al., 1990). NF action is accompanied by a series of signal transduction processes inside root cells. Intense research has focused on elucidating NF signaling, which plays an essential role in nodule organogenesis (Dénarié et al., 1996; Oldroyd and Downie, 2008; Masson-Boivin et al., 2009). Genetic dissection of nodulation in legumes such as *Medicago truncatula* and *Lotus japonicus* has led to the discovery of NF receptors and several key players in the NF signaling pathway (Oldroyd and Downie, 2004, 2006). Symbiosis receptor-like kinase (SymRK) is required for symbiotic signal transduction upon stimulation of root cells by microbial signaling molecules (Endre et al., 2002; Stracke et al., 2002). However, the exact biochemical functions and regulatory mechanisms of SymRK remain unknown.

Recent studies by various groups have identified several candidate proteins that interact with SymRK and are required for root nodule symbiosis, including *M. truncatula* 3-hydroxy-3-methylglutaryl CoA reductase 1 (MtHMGR1) (Kevei et al., 2007) Symbiotic Remorin 1 (SYMREM 1) (Lefebvre et al., 2010) Plant U-box Protein 1 (PUB1) (Vernié et al., 2016), *L. japonicus* SymRK interacting protein 1 (SIP1) (Zhu et al., 2008), SymRK interacting protein 2 (SIP2) (Chen et al., 2012), SymRK-interacting E3 ligase (SIE3) (Yuan et al., 2012) SEVEN IN ABSENTIA 4 (SINA4) (Den Herder et al., 2012), and Nod factor receptor 5 (NFR5) (Antolin-Llovera et al., 2014). These studies suggest that SymRK forms protein complexes with key regulatory proteins of downstream cellular responses and participates in different signaling pathways. Symbiotic Remorin 1 (SYMREM 1) from *M. truncatula* interacts with various symbiotic receptor kinases including NFP/NFR5, LYK3/NFR1, and DMI2/SymRK and may act as a scaffold protein for the assembly of signaling complexes involved in rhizobial infection (Lefebvre et al., 2010).

Several E3 ligases have been shown to be regulated and/or play a role in bacterial infection or nodulation (Vinardell et al., 2003; Shimomura et al., 2006; Den Herder et al., 2008; Kiss et al., 2009; Yano et al., 2009; Mbengue et al., 2010; Den Herder et al., 2012; Yuan et al., 2012; Cai et al., 2018; Tsikou et al., 2018). E3 ligases, which are essential for protein ubiquitination, fall into two classes: the RING (Really Interesting New Gene)-finger family (Petroski and Deshaies, 2005) and the HECT (homologous to E6-AP carboxy terminus) family (Zheng, 2003). Based on the combination of cysteine (C) and histidine (H) residues in the RING domain, RING finger-related E3 ligases are divided into the C3HC4, C2H2C4, C3H2C3, and C4H4 classes (Petroski and Deshaies, 2005; Deshaies and Joazeiro, 2009). These proteins usually form homodimers or heterodimers and are thus referred to as dimeric E3 ubiquitin ligases (Bellon et al., 1997; Li et al., 2006; Plechanovová et al., 2011).

In this study, we demonstrate that SIE3 is a novel plant dimeric E3 ubiquitin ligase whose disulfide linkage at Cys266 plays key roles in the formation and symbiosis function of SIE3 homodimers. Moreover, we demonstrate that the SIE3 E3 ligase interacts with the transcription factor SIP1.

## Results

### SIE3 Interacts With SIP1 in Yeast Cells

**FIGURE 1:**
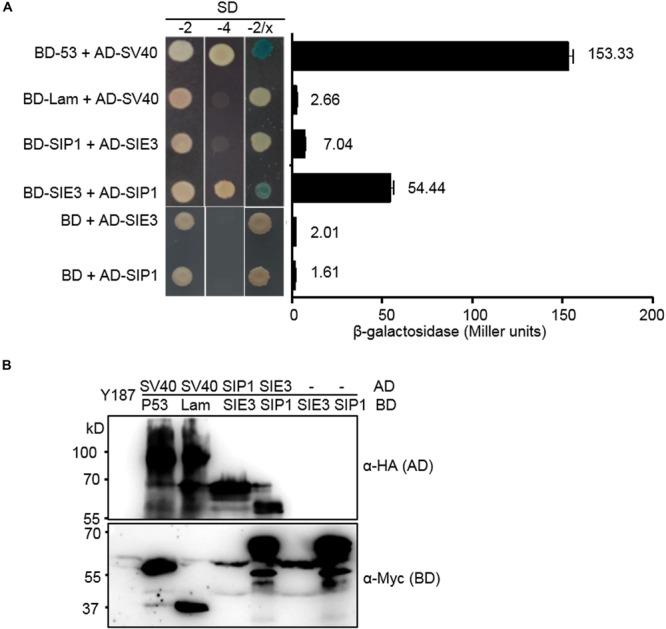
SIE3 interacts with SIP1 in yeast cells. **(A)** Interaction between SIE3 and SIP1 in yeast cells. Proteins were fused with the Gal4 DNA binding domain (BD) in pGBKT7 or with its activation domain (AD) in pGADT7. Yeast cells harboring the constructs were maintained on SD/-Trp-Leu medium (SD-2) and selected for protein-protein interactions on SD/-Trp-Leu-His-Ade (SD-4) or SD-2/X-gal medium. The strength of the interaction was evaluated based on β-galactosidase activity (Miller units). At least three biological replicates were performed, and the data are presented as the mean ± SD. The combination p53/SV40 served as a positive control, and Lam/SV40, BD-SIE3/AD and BD-SIP1/AD served as negative controls. **(B)** Immunoblot analysis of protein levels in yeast cells. Anti-HA monoclonal antibody was used to detect the expression levels of HA-tagged proteins (AD-SV40, AD-SIE3, and AD-SIP1). Anti-Myc monoclonal antibody was used to detect the levels of Myc-tagged proteins (BD-53, BD-Lam, BD-SIE3, and BD-SIP1).

We previously demonstrated that SymRK interacts with both SIP1, an ARID-type transcription factor and SIE3, a RING-type E3 ubiquitin ligase in *L. japonicus* (Zhu et al., 2008; Yuan et al., 2012). Here, we investigated whether SIP1 interacts with SIE3. To test this hypothesis, we conducted a yeast two-hybrid (Y2H) assay to examine the interaction between SIP1 and SIE3. As shown in Figure 1, yeast cells containing BD-SIE3/AD-SIP1 or AD-SIE3/BD-SIP1 grew on quadruple dropout SD medium and had higher β-galactosidase activity than the negative controls (Figure 1A). The expression of recombinant proteins in yeast was confirmed by immunoblot analysis using anti-hemagglutinin (HA) or anti-Myc monoclonal antibodies (Figure 1B). These results indicate that SIE3 associates with SIP1 in yeast cells.

### Interaction of SIE3 With SIP1 *in planta*

**FIGURE 2:**
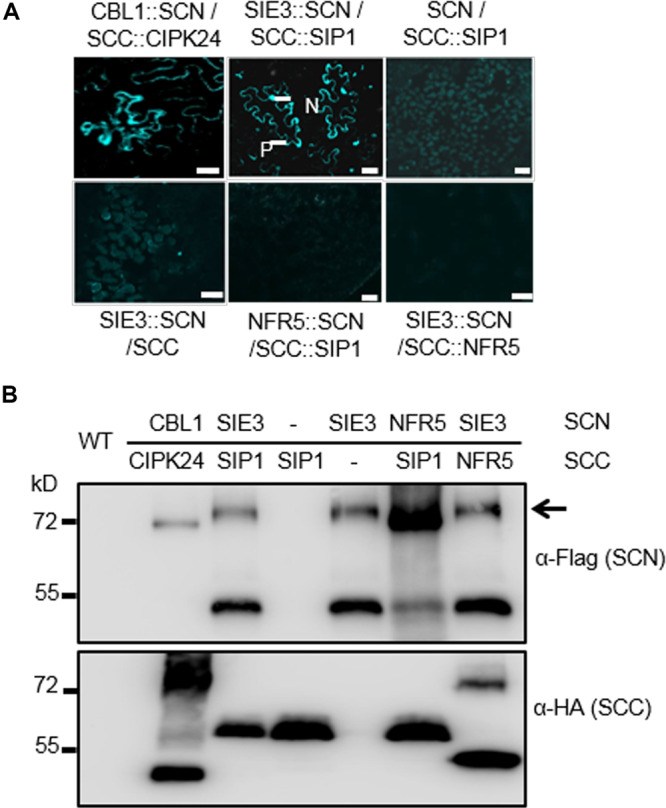
SIE3 interacts with SIP1 in *planta*. **(A)** BiFC assay of the interaction of SIE3 with SIP1 *in planta*. *N. benthamiana* leaves were co-transfected with *Agrobacterium* cells expressing SCC::SIP1 and SIE3::SCN. The combination of SCC::CBL24 and CBL1::SCN served as a positive control, while the combinations of SCN and SCC::SIP1, SIE3::SCN and SCC, NFR5::SCN and SCC::SIP1, and SIE3::SCN and SCC::NFR5 were used as negative controls. Five samples were observed for each combination. N, nucleus; P, plasma membrane. Bar = 30 μm for SCC::CBL24 and CBL1::SCN and 20 μm for the remaining samples. **(B)** Immunoblot analysis of proteins expressed in *N. benthamiana* leaves. The BiFC and immunoblot analyses were performed at least three biological replicates. Anti-Flag monoclonal antibody was used to detect the expression levels of Flag-tagged proteins (SCN::CBL1, SCN::SIE3, and SCN::NFR5). Anti-HA monoclonal antibody was used to detect the expression levels of HA-tagged proteins (CIPK24::SCC, SIP1::SCC, and NFR5::SCC). The black arrow indicates the Flag-SIE3 dimer.

We performed bimolecular fluorescence complementation (BiFC) assays to investigate whether the SIE3/SIP1 interaction occurs *in planta* using *Nicotiana benthamiana* leaf cells. SIP1 was fused to the split C-terminus of CFP (SIP1::SCC), while SIE3 was fused with the split N-terminus of this protein (SCN::SIE3). We analyzed *N. benthamiana* leaf epidermal cells 2–5 days after infiltration with *Agrobacterium tumefaciens* harboring these constructs. Strong fluorescent signals from leaves expressing both SIP1::SCC and SCN::SIE3 were observed in the plasma membrane and nucleus. This pattern was similar to that of leaves expressing the positive control proteins *Arabidopsis* CALCINEURIN B-LIKE (CBL) and CBL-INTERACTING PROTEIN KINASE 24 (CIPK24) (Figure 2A; Waadt et al., 2008). In the negative controls, where SIP1::SCC and SCN::SIE3 were expressed separately or SCN::NFR5 and SCC::SIP1, and SCN::SIE3 and SCC::NFR5 were expressed, no fluorescent signals were observed (Figure 2A). The expression levels of recombinant proteins in *N. benthamiana* leaves were confirmed by immunoblot analysis using anti-HA or anti-Flag monoclonal antibodies (Figure 2B). These results indicate that SIP1 and SIE3 interact with each other in *planta*.

### SIE3 Dimerization

**FIGURE 3:**
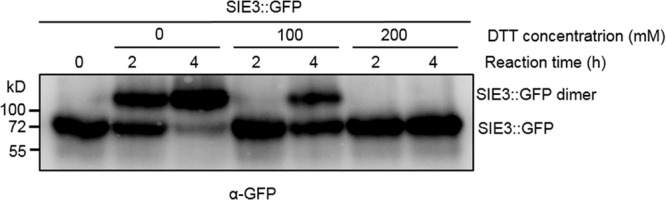
Effect of DTT on SIE3 dimerization. Total proteins were extracted from *N. benthamiana* leaves expressing SIE3::GFP in native buffer. The SIE3::GFP extract was incubated in the presence of 10 μM ATP at 4°C with gentle shaking. A final concentration of 0, 100, or 200 mM DTT was added to the reaction. Reactions were stopped by adding SDS sample loading buffer without DTT, and the products were analyzed with anti-GFP antibody. All experiments were performed with at least three biological replicates.

When SIE3 was expressed *in planta*, we observed a high molecular mass (Figure 2B). Based on its size, we reasoned that this band might correspond to SIE3 homodimer. To examine whether reduced thiol compounds would affect SIE3 dimerization, we tested whether SIE3 monomer could form homodimers with or without the reducing agent DTT (McGinnes and Morrison, 1998) in the reaction (Figure 3). In buffer lacking DTT, the majority of band signals were detected above the 130 kDa position, representing potential SIE3 dimers in the reaction (Figure 3, lanes 2–3). In buffer supplemented with 100 mM DTT, nearly equal amounts of upper (about 137 kDa) and lower bands (about 68.5 kDa) were detected after 4 h of incubation (Figure 3, lane 5), while in the presence of 200 mM DTT, only a band corresponding the monomeric form (about 68.5 kDa) was observed (Figure 3, lanes 6–7). These results suggest that the upper band corresponds to the SIE3 homodimer and the lower band to the monomer, and that the presence of DTT in the reaction mixture helped to stabilize the SIE3 monomer.

### Cysteine 266 of SIE3 Is Required for Homodimerization

**FIGURE 4:**
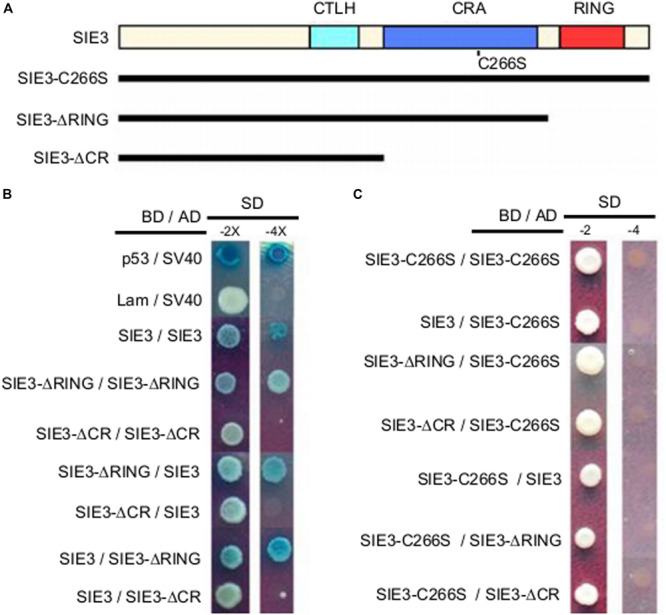
Identification of the peptide domain required for SIE3 dimer formation in yeast cells. **(A)** Functional domains of SIE3 and its mutant constructs. CTLH (for C-terminus to LisH), CRA (for CT11-RanBPM), RING (for Really Interesting New Gene). SIE3-ΔRING lacked the RING domain, while SIE3-ΔCR lacked both the CRA-RanBPM and RING domains. In SIE3-C266S, Cys266 was replaced with serine. **(B)** Interaction between SIE3 and SIE3 deletion mutants in yeast cells. Proteins were fused with the Gal4 DNA binding domain (BD) in pGBKT7 or with its activation domain (AD) in pGADT7. Yeast cells harboring the constructs were maintained on SD/-Trp-Leu medium (SD-2) and selected for protein-protein interactions on SD/-Trp-Leu-His-Ade/X-gal (SD-4 or SD-4/X-gal) medium. The combinations p53/SV40 and Lam/SV40 served as positive and negative controls, respectively. **(C)** Interactions between SIE3-C266S and SIE3 deletion mutants in yeast cells. For the yeast two-hybrid assay, at least three independent biological replicates were performed.

To pinpoint the peptide domains required for homodimerization of SIE3, we generated deletions of *SIE3* cDNA (Figure 4A) and used the Y2H assay to test the interactions between them. The SIE3 deletion mutant containing the domains C-terminal region to LisH (CTLH) and CRA-RanBPM but lacking the C-terminal RING domain (SIE3-ΔRING) interacted with itself, suggesting that the RING domain is not essential for homodimerization (Figure 4B). When both the CRA-RanBPM and RING domains were removed (SIE3-ΔCR), no self-interaction of SIE3 was observed (Figure 4B). Taken together, these results suggest that the CRA-RanBPM domain is essential for the self-interaction of SIE3.

**FIGURE 5:**
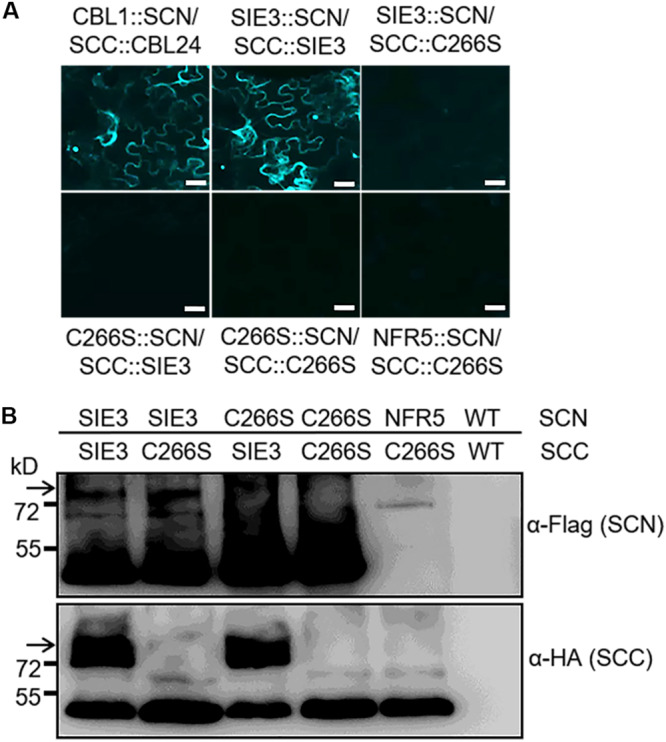
BiFC assay of the interactions of SIE3 with itself or SIE3-C266S *in planta*. **(A)** *N. benthamiana* leaves were co-transfected with *Agrobacterium* cells expressing SIE3::SCN and SCC:: SIE3, SIE3::SCN and SCC::C266S, C266S::SCN and SCC::SIE3, or C266S::SCN and SCC::C266S. The combination of SCC::CBL24 and CBL1::SCN served as a positive control, while the combination of NFR5::SCN and SCC::C266S was used as a negative control. Bar = 30 μm. **(B)** Immunoblot analysis of proteins expressed in *N. benthamiana* leaves. Anti-Flag monoclonal antibody was used to detect the expression of Flag-tagged proteins (SCN::SIE3, SCN::SIE3-C266S, and SCN::NFR5). Anti-HA monoclonal antibody was used to detect the expression of HA-tagged proteins (SIE3::SCC and SIE3-C266S::SCC). White arrows indicate the SIE3 dimer. The BiFC and immunoblot analyses were performed at least three biological replicates.

Since DTT destroyed the dimerization of SIE3 (Figure 3), we hypothesized that the disulfide bonds whose formation is mediated by cysteine residues might be required for SIE3 homodimerization. Among the seven cysteine residues of SIE3, six are located in the RING-finger domain, which was not necessary for the SIE3-SIE3 interaction in yeast cells. The remaining cysteine reside (Cys266) is present in the CRA-RanBPM domain, which was essential for the self-interaction of SIE3. We therefore hypothesized that Cys266 might be the key residue controlling SIE3 homodimerization. In fact, the SIE3 form with Cys266 substituted to serine (SIE3-C266S) was unable to form homodimers in yeast or *N. benthamiana* leaf cells (Figures 4C, 5). This result, together with the observation that the SIE3 dimer was sensitive to DTT (Figure 3), indicates that the dimerization of SIE3 is mediated by the formation of a disulfide bond via Cys266.

**FIGURE 6:**
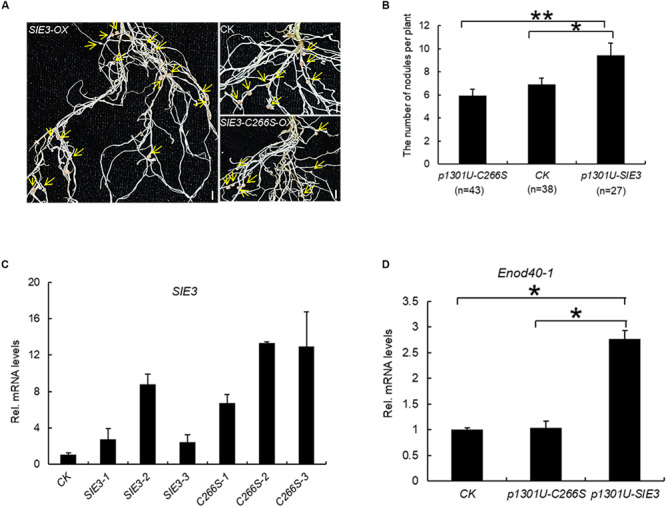
Effect of *SIE3-C266S* overexpression on nodulation. **(A)** Nodulation phenotypes of WT, *SIE3-OX*, and *SIE3-C266S-OX* hairy roots. Transgenic hairy roots were produced using *A. rhizogenes* cells carrying the *SIE3-OX* or *SIE3-C266S-OX* construct. Transgenic hairy roots transformed with the empty vector (p1301U) served as the control. The hairy roots were inoculated with *M. loti* and grown in nitrogen fertilizer-free soil to induce nodulation. Three weeks after rhizobial inoculation, one representative independent transgenic hairy root (with nodules) per sample was photographed; yellow arrows indicate nodules. Bars, 5 mm. **(B)** Number of nodules per plant of transgenic hairy roots with altered *SIE3* and *SIE3-C266S* transcript levels. Transgenic hairy roots expressing empty vector (pU1301) served as a control. Two large-scale experiments were conducted, and the total number of independent transgenic plants per sample is indicated in parentheses. Data represent the mean ± SE. **(C)** Analysis of *SIE3* transcript levels in control (control), *SIE3-OX* (*SIE3-OX*-1, *SIE3-OX*-2, *SIE3-OX*-3), and *SIE3-C266S-OX* (*SIE3-C266S-OX*-1, *SIE3-C266S-OX*-2, *SIE3-C266S-OX*-3) hairy roots. Total RNA isolated from individual root systems was used for qPCR. Relative expression levels of *SIE3* transcripts in *SIE3-OX* and *SIE3-C266S-OX* hairy roots were calculated with reference to that of control hairy roots. **(D)** qPCR analysis of *Enod40-1* transcript levels in control and transgenic hairy roots. Total combined RNA isolated from the root systems (including small nodules) of multiple independent transgenic plants was used for qPCR analysis. Relative expression levels of *Enod40-1* in transgenic hairy roots were calculated with reference to that of control hairy roots, at least three independent biological replicates and three technical replicates for each sample. The data are presented as the mean ± SD **(C,D)**. “*”and “**” indicate significant differences between samples (*t*-test, *p* < 0.05 or *p* < 0.01, respectively).

The results of this work indicate that SIE3 forms a homodimer. We asked whether this homodimer is required for its function in the nodule symbiosis. We expressed *SIE3* and *SIE3-C266S* under the control of the maize *Ubiquitin* promoter (*SIE3-OX* and *SIE3-C266S-OX*) in transgenic hairy roots of *L. japonicus* and scored the nodulation phenotypes 3 weeks after inoculation with *Mesorhizobium loti* MAFF303099 expressing β-galactosidase (lacZ), a constitutive marker used to observe rhizobial cells (Tansengco et al., 2003). Compared to control transgenic roots, significantly more nodules were produced in *SIE3-OX* hairy roots, whereas the number of nodules in *SIE3-C266S-OX* hairy roots was similar to that of the transgenic hairy roots expressing empty vector (control) (Figures 6A,B). Quantitative PCR (qPCR) analysis indicated that the expression level of the transgene in *SIE3-OX* or *SIE3-C266S-OX* hairy roots was 3–15-fold higher compared to the control (Figure 6C). qPCR analysis of *ENOD40-1*, an early nodulin gene implicated in the processes of rhizobial infection, nodule initiation, and subsequent organogenesis (Kumagai et al., 2006) was performed. The results showed that *ENOD40-1* was expressed at higher levels in *SIE3-OX* hairy roots than in the control (Figure 6D). These data indicate that cysteine-266 of SIE3 is essential for its function in promoting nodulation in *L. japonicus*.

## Discussion

SymRK is an essential player in bacterial entry and is required for nodulation in legumes (Stracke et al., 2002; Radutoiu et al., 2003; Yoshida and Parniske, 2005). Various proteins that interact with SymRK have recently been identified. However, how NF signals are transduced downstream of SymRK remains unclear. To better understand the relationships between SymRK-interacting proteins, we investigated the interaction between SIP1 and SIE3, two SymRK-interacting proteins. Our results demonstrate that SIE3 and SIP1 interact with each other both in yeast and *in planta*. Our results also demonstrate that SIE3 can form homodimers through a disulfide linkage at Cys266 and that a mutation at Cys266 of SIE3 abolished its role in symbiosis.

RING domain E3 ubiquitin ligases are modular proteins with RING and substrate-binding domains. Mammalian RING finger protein 4 (RNF4) is a dimeric RING E3 ligase. The RING domain of RNF4 is responsible for ubiquitin transfer and its dimerization, which are essential for E3 ligase activity (Liew et al., 2010). During the ubiquitination reaction, the activated ubiquitin tag is covalently linked to a cysteine (Cys) residue of E2 conjugase via a thioester bond. In the RNF4 dimer, one subunit is bound to E2 conjugase while the other subunit engages the thioester-linked ubiquitin (Plechanovová et al., 2011). Thus, the dimerization of RNF4 is required for the transfer of ubiquitin from E2 conjugase to the substrate. Dimerization and similar catalytic mechanisms have been observed for several other RING-type E3 ubiquitin ligases, including cIAP2 (Cellular inhibitor of apoptosis protein 2) (Mace et al., 2008), TRAF6 (TNF receptor-associated factor 6) (Vander Kooi et al., 2006; Yin et al., 2009), MDM2-MDMX19, BARD1 [breast cancer susceptibility gene 1 (BRCA1)-BRCA1-associated RING domain protein 1] (Brzovic et al., 2001) Ring1b-Bmi1 (B-cell-specific Moloney murine leukemia virus integration site 1) (Buchwald et al., 2006) and *Arabidopsis* SINA5 (Xie et al., 2002). However, little is known about the role of E3 dimerization in legume plants.

*L. japonicus* SIE3 interacted with itself in yeast cells and *N. benthamiana* leaves (Figures 2B, 4, 5). The SIE3 monomer might have become a homodimer during the reaction process (Figure 3). It appears that SIE3 primarily exists as a homodimer in plants. The ubiquitination of SymRK is mediated by SIE3 and the RING domain of SIE3 appears to be essential for its interactions with SymRK (Yuan et al., 2012). However, unlike the above-mentioned E3 proteins, the CRA-RanBPM domain of SIE3 rather than its RING domain is essential for SIE3 dimerization (Figure 4B). Thus, SIE3 might represent a novel plant dimeric E3 ubiquitin ligase.

Protein dimerization can occur through the formation of disulfide bonds and is sensitive to DTT (McGinnes and Morrison, 1998). DTT has a strong effect on the formation of SIE3 homodimers (Figure 3). The CRA-RanBPM domain of SIE3 is essential for its homodimerization (Figure 4B). The CRA-RanBPM domain contains a single cysteine residue (C266). The replacement of this residue with serine (SIE3-C266S) blocked the formation of SIE3 homodimers in yeast and *N. benthamiana* leaves (Figures 4C, 5), further implying that SIE3 dimers form through a disulfide linkage at Cys266. In addition, significantly more nodules were produced in *SIE3OX* hairy roots than in the control (Yuan et al., 2012; Figures 6A,B), while there were no more or even fewer nodules in *SIE3-C266SOX* hairy roots than in the control (Figures 6A,B). In *Arabidopsis*, the expression of the dominant-negative SINA5-C49S affected the endogenous E3 ligase activity of SINA5, thereby downregulating the ubiquitination of the NAC1 (NUCLEUS ACCUMBENS-ASSOCIATED 1) transcription factor and auxin signaling in plant cells (Xie et al., 2002). Similarly, the presence of SIE3-C266S perhaps affected the ability of endogenous SIE3 to form homodimers, thereby downregulating the normal biological functioning of SIE3.

Previously, our laboratory has shown that SIP1 can interact with SymRK (Zhu et al., 2008). In addition, SIE3 can interact with SymRK and mediate ubiquitination of SymRK in *L. japonicus* (Yuan et al., 2012). In this report, SIE3 can interact with SIP1 in yeast and *in planta* (Figure 1 and Figure 2). It is possible that SIP1, SIE3, and SymRK form a trimeric complex, in which the E3 ligase SIE3 is bound to two protein substrates. In the gibberellic acid (GA) signaling pathway, the interaction between the GA receptor GID1 and its downstream component DELLA protein can help SCF^*SLY1*/*GID2*^ to gain its DELLA protein specificity (Schwechheimer, 2008). In a proposed model for CUL7-mediated TBC1D3 oncoprotein degradation in human, a third protein, Fbw8, has been shown to interact with CUL7 and also to be required for TBC1D3 degradation (Kong et al., 2012). However, whether a trimeric complex of SIE3, SymRK, and SIP1 exists in developing nodules during the establishment of the nodule symbiosis awaits further experimental confirmation.

In summary, SIE3 appears to represent a novel dimeric E3 ubiquitin ligase in plants. The cysteine 266 residue was found to be essential for SIE3 dimerization and for promoting nodulation in transgenic hairy roots of *L. japonicus*. SIE3, SIP1, and SymRK, have previously been shown to play important roles in the transduction of NF signals and during early nodule organogenesis. The current results shed new light on the complex relationships among SymRK, SIE3, and SIP1 and provide a foundation for further investigating the regulatory mechanisms of the SymRK-mediated signaling pathway, as well as the biological function of E3 ligase dimerization in nodule organogenesis.

## Materials and Methods

### Plant Materials and Growth Conditions

*N. benthamiana* plants were grown in a growth chamber at 22°C and 40–70% relative humidity (RH) under a 16 h light/8 h dark photoperiod for about 4–6 weeks before infiltration with *A. tumefaciens* strain EHA105. After infiltration, plants were kept under the same growth conditions. Wild-type (WT) plants of *L. japonicus* (Handberg and Stougaard, 1992). *”Miyakojima* MG-20” were used for hairy root transformation. Seeds were scarified by immersion in concentrated H_2_SO_4_ for 8 min before surface sterilization in 2% NaClO plus 0.1% Tween-20 for 20 min. Seeds were plated on 1/2 MS medium supplemented with 0.8% agar for germination at 28°C in the dark for 2 days and then transferred to a growth chamber with a 16/8 h light/dark cycle at 23°C.

### Protein-Protein Interaction in Yeast Cells

Full-length cDNA of *SIE3*, *SIE3-ΔRING*, *SIE3-ΔCR*, and *SIE3-C266S* cDNAs were amplified by PCR and inserted into *Nde*I/*EcoR*I sites of pGBKT7 or pGADT7 (Yuan et al., 2012). AD-SIP1 and BD-SIP1 have been described in previous papers (Zhu et al., 2008; Wang et al., 2013). Small-scale mating tests were performed to examine protein-protein interactions. After mating, yeast cells harboring the constructs were maintained on SD/-Trp-Leu medium (SD-2) and selected for protein-protein interaction on SD/-Trp-Leu-His-Ade (SD-4) or SD-2/X-gal. Colonies on SD/-Leu-Trp-His-Ade plates were transferred to the SD/-Leu-Trp/X-gal plate for further verification using β-galactosidase assay (Clontech). Proteins were extracted from yeast expressing recombinant proteins using yeast cracking buffer (40 mM Tris-HCl pH = 6.8), 8 M urea, 5% (w/v) SDS, 0.1 mM EDTA, 5% β-mercaptoethanol, 10 mM DTT, 0.4% (w/v) Bromophenol blue, Protease inhibitor solution (Roche), 1 mM phenylmethylsulfonyl fluoride. The cracking buffer was prewarmed to 60°C, and cell pellets were thawn quickly by separately resuspending each sample in the prewarmed cracking buffer. Each sample was transferred to a 1.5 mL microcentrifuge tube containing glass beads, and heated at 70°C for 10 min, vortexed vigorously for 2 min, and centrifuged at 14,000 rpm for 5 min. The supernatants were then transferred to 1.5 mL microcentrifuge tubes and kept as first supernatants. The pellets were boiled for 3–5 min, vortexed vigorously for 1 min, and centrifuged at 14,000 rpm for 5 min. These second supernatants were then combined with the first supernatants. The samples were briefly boiled and loaded on a SDS-PAGE gel. Anti-HA monoclonal antibody (EarthOx, 1:1000) was used to detect the expression levels of HA-tagged proteins AD-SIE3 (60 kD) and AD-SIP1 (63.2 kD); Anti-Myc monoclonal (EarthOx, 1:1000) antibody was used to detect the expression levels of Myc-tagged proteins BD-SIE3 (63.2 kD) and BD-SIP1 (66.4 kD). Tests were performed with at least three biological replicates.

### β-Galactosidase Assay

Yeast cells grown in liquid selection media were measured at OD_600_ and centrifuged for 30 s at 13,000 rpm. Cells were washed twice with Z-buffer (60 mM Na_2_HPO_3_, 40 mM NaH_2_PO_3_, 10 mM KCl, 1.0 mM MgSO_4_, pH = 7.0), and resuspended in 100 μL of Z-buffer, followed by permeabilization by three freeze-thaw cycles in liquid nitrogen and 37°C water bath. After centrifugation, cell extracts were added to 0.7 mL of Z-buffer containing 50 mM β-mercaptoethanol and 160 μL of ONPG (O-nitrophenyl β-D-galactopyranoside, 4 mg/mL in Z-buffer). After incubation at 30°C until the yellow color appeared, the reaction was terminated by the addition of 0.4 mL of 1.0 M Na_2_CO_3_. The reaction mixture was centrifuged for 10 min at 13,000 rpm to remove cell debris. β-Galactosidase activity in the supernatant was measured at OD_420_, and expressed in Miller units (Griffith and Wolf, 2002). All reactions were performed with at least three biological replicates.

### BiFC Experiments

The full-length cDNA of *SIP1*, *SIE3*, and *SIE3-C266S* were cloned into the *Spe*I/*Kpn*I site of pSCYCE-R, which contains a HA-tag (Waadt et al., 2008) to obtain SIP1::SCC, SIE3::SCC and SIE3-C266S::SCC fusions. The full-length cDNA of *SIE3* and *SIE3-C266S* were cloned into the *Bam*HI/*Xho*I site of pSCYNE, which contains a FLAG-tag (Waadt et al., 2008) to obtain SIE3::SCN and SIE3-C266S::SCN. Plasmids were introduced into *A. tumefaciens* strain EHA105 cells by electroporation. *Agrobacterium* cells containing plasmids were grown in liquid medium (LB broth with 50 μM kanamycin, pH = 7.0, 10 mM MES, pH = 5.7, 40 μM acetosyringone) were pelleted and resuspended in the infiltration buffer (10 mM MgCl_2_, 200 μM acetosyringone). *Agrobacterium* strains containing different plasmids were mixed to a final OD_600_ of 0.75 for each strain, and then mixed with the *Agrobacterium* strain containing the gene-silencing suppressor p19 at a final OD_600_ of 1.0 in a volume ratio of 1:1. The mixture of *Agrobacterium* strains was incubated at room temperature for 4 h, and used for infiltration into the leaves of 4–6 week-old *N. benthamiana* plants using a 1 mL syringe. Cyan fluorescence was observed 3–5 days after infiltration of leaf cells expressing these proteins under the confocal microscope OLYMPUS BX61WI equipped with a CFP filter set (excitation/emission wavelengths of 405/477 nm). The BiFC analyses were performed with at least three independent biological replicates.

### Protein Extraction and Immunoblot Analysis

Proteins were extracted from *N. benthamiana* leaves expressing recombinant proteins using native extraction buffer 1 (NB1) and denaturing buffer (DB) as described (Yuan et al., 2012). NB1 contained 50 mM Tris-MES, pH = 8.0, 0.5 M sucrose, 1 mM MgCl_2_, 10 mM EDTA, 5 mM DTT, and protease inhibitor cocktail CompleteMini tablets (Roche). DB contained 50 mM Tris-HCl, pH = 7.5, 150 mM NaCl, 0.1% NP-40, 4 M urea, and protease inhibitor cocktail CompleteMini tablets. The leaf areas surrounding the infiltrated sites were harvested, and ground in liquid nitrogen, then 1 mL of each leaf powder was filled in a 2 mL centrifuge tube. Leaf powders were resuspended in extraction buffer on ice. Total extracts were centrifuged at 13,000 rpm at 4°C for 30 min. Supernatants were subjected to Western blot analysis. Same quantity of leaf powders were extracted (corresponding to same quantity of total leaf protein) and used to normalize loading the SDS-PAGE gels, and Ponceau S staining of the Rubisco band on the membrane was used to assess the amounts of samples on each lane on the SDS-PAGE gels.

The sources and dilutions of antibodies used in the experiments were as follows: anti-HA antibody (Sigma, 1:5000; EarthOx, 1:1000), anti-Flag antibody (EarthOx, 1:1000), anti-GFP antibody (Abmart, 1:1000). Proteins were separated by SDS-PAGE in a 10 or 12% acrylamide gel and semi-dry electroblotted to nitrocellulose membrane (Hybond-C, Amersham)^1^ at 25 V for 40 min. The membrane was blocked with PBS containing 5% skimmed milk powder for 1 h at room temperature or overnight at 4°C. The membrane was then incubated first with primary antibody in PBS containing 3% skimmed milk for more than 1 h at room temperature, and then with secondary antibody diluted in TBS containing 3% skimmed milk for 1 h at room temperature. Bands were visualized with the Millipore chemiluminescent HRP substrate kit. Anti-HA monoclonal antibody was used to detect the expression levels of HA-tagged proteins SIP1::SCC (58 kD), SIE3:: SCC (52.8 kD), and SIE3-C266S::SCC (52.8 kD). Anti-Flag monoclonal antibody was used to detect the expression levels of Flag-tagged proteins SCN::SIE3 (52.7 kD) and SCN::SIE3-C266S (52.7 kD). Anti-GFP monoclonal antibody was used to detect the expression levels of SIE3::GFP (68.5 kD). All experiments were performed at least three independent biological replicates.

### Overexpression of SIE3 and SIE3-C266S by Hairy Root Transformation

The full-length CDS of *SIE3* and *SIE3-C266S* were cloned into the *Kpn*I/*BamH*I site of p1301U to obtain pMUb: *SIE3* and pMUb: *SIE3-C266S*, respectively. *A. rhizogenes* strain LBA1334 cells carrying pMUb: *SIE3* or pMUb: *SIE3-C266S* were used to induce hairy root formation in wild-type *L. japonicus* “MG-20” using a procedure as described previously (Yuan et al., 2012). Nodulation phenotypes of transgenic hairy roots were scored 3 weeks after inoculation with *M. loti* MAFF303099. Transgenic hairy roots expressing the empty vector (p1301U) were used as a control. The transgenic hairy roots (with nodules) were photographed; the mean values of nodule number and Student’s *t*-tests were performed using software SPSS Statistics 17.0. The expression level of *SIE3* and *Enod40-1* in *SIE3*-OX or *SIE3-C266S*-OX hairy roots was determined by qPCR using the primers and the procedure as described previously (Yuan et al., 2012).

## Data Availability Statement

All datasets generated for this study are included in the article/Supplementary Material.

## Author Contributions

SY, ZZ, and ZH designed this work and wrote the manuscript. YF and SY performed most of the experiments. PW, WF, LP, HZ, YC, and XZ contributed substantially to the completion of this work.

## Conflict of Interest

The authors declare that the research was conducted in the absence of any commercial or financial relationships that could be construed as a potential conflict of interest.

## Footnotes

1 http://www6.gelifesciences.com/

## References

[B1] Antolin-Llovera M. Ried M. K. Parniske M. (2014). Cleavage of the SYMBIOSIS RECEPTOR-LIKE KINASE ectodomain promotes complex formation with Nod factor receptor 5. *Curr. Biol.* 24 422–427. 10.1016/j.cub.2013.12.053 24508172

[B2] Bellon S. F. Rodgers K. K. Schatz D. G. Coleman J. E. Steitz T. A. (1997). Crystal structure of the RAG1 dimerization domain reveals multiple zinc-binding motifs including a novel zinc binuclear cluster. *Nat. Struct. Biol.* 4 586–591. 10.1038/nsb0797-586 9228952

[B3] Brzovic P. S. Rajagopal P. Hoyt D. W. King M.-C. Klevit R. E. (2001). Structure of a BRCA1–BARD1 heterodimeric RING–RING complex. *Nat. Struct. Biol.* 8 833–837. 10.1038/nsb1001-833 11573085

[B4] Buchwald G. van der Stoop P. Weichenrieder O. Perrakis A. van Lohuizen M. Sixma T. K. (2006). Structure and E3-ligase activity of the Ring–Ring complex of polycomb proteins Bmi1 and Ring1b. *EMBO J.* 25 2465–2474. 10.1038/sj.emboj.7601144 16710298 PMC1478191

[B5] Cai K. Yin J. Chao H. Ren Y. Jin L. Cao Y. (2018). A C3HC4-type RING finger protein regulates rhizobial infection and nodule organogenesis in Lotus japonicus. *J. Integr. Plant Biol.* 60 878–896. 10.1111/jipb.12703 30047576

[B6] Chen T. Zhu H. Ke D. Cai K. Wang C. Gou H. (2012). A MAP kinase kinase interacts with SymRK and regulates nodule organogenesis in *Lotus japonicus*. *Plant Cell* 24 823–838. 10.1105/tpc.112.095984 22353370 PMC3315249

[B7] Den Herder G. De Keyser A. De Rycke R. Rombauts S. Van de Velde W. Clemente M. R. (2008). Seven in absentia proteins affect plant growth and nodulation in *Medicago truncatula*. *Plant Physiol.* 148 369–382. 18599652 10.1104/pp.108.119453 PMC2528092

[B8] Den Herder G. Yoshida S. Antolín-Llovera M. Ried M. K. Parniske M. (2012). Lotus japonicus E3 ligase SEVEN IN ABSENTIA4 destabilizes the symbiosis receptor-like kinase SYMRK and negatively regulates rhizobial infection. *Plant Cell* 24 1691–1707. 22534128 10.1105/tpc.110.082248 PMC3398572

[B9] Dénarié J. Debellé F. Promé J.-C. (1996). Rhizobium Lipo-Chitooligosaccharide Nodulation Factors: signaling molecules mediating recognition and morphogenesis. *Ann. Rev. Biochem.* 65 503–535. 10.1146/annurev.bi.65.070196.002443 8811188

[B10] Deshaies R. J. Joazeiro C. A. P. (2009). RING domain E3 ubiquitin ligases. *Annu. Rev. Biochem.* 78 399–434. 10.1146/annurev.biochem.78.101807.093809 19489725

[B11] Endre G. Kereszt A. Kevei Z. Mihacea S. Kalo P. Kiss G. B. (2002). A receptor kinase gene regulating symbiotic nodule development. *Nature* 417 962–966. 10.1038/nature00842 12087406

[B12] Griffith K. L. Wolf R. E. (2002). Measuring β-Galactosidase Activity in bacteria: cell growth, permeabilization, and enzyme assays in 96-well arrays. *Biochem. Biophys. Res. Co* 290 397–402. 10.1006/bbrc.2001.6152 11779182

[B13] Handberg K. Stougaard J. (1992). Lotus japonicus, an autogamous, diploid legume species for classical and molecular genetics. *Plant J.* 2 487–496. 10.1111/j.1365-313X.1992.00487.x

[B14] Kevei Z. Lougnon G. Mergaert P. Horváth G. V. Kereszt A. Jayaraman D. (2007). 3-Hydroxy-3-Methylglutaryl Coenzyme A reductase1 interacts with NORK andis crucial for nodulation in *Medicago truncatula*. *Plant Cell* 19 3974–3989. 10.1105/tpc.107.053975 18156218 PMC2217646

[B15] Kiss E. Oláh B. Kaló P. Morales M. Heckmann A. B. Borbola A. (2009). LIN, a novel type of U-Box/WD40 protein, controls early infection by rhizobia in legumes. *Plant Physiol.* 151 1239–1249. 10.1104/pp.109.143933 19776163 PMC2773052

[B16] Kong C. Samovski D. Srikanth P. Wainszelbaum M. J. Charron A. J. Liu J. (2012). Ubiquitination and degradation of the hominoid-specific oncoprotein TBC1D3 is mediated by CUL7 E3 ligase. *PLoS One* 7:e46485. 10.1371/journal.pone.0046485 23029530 PMC3459922

[B17] Kumagai H. Kinoshita E. Ridge R. W. Kouchi H. (2006). RNAi Knock-down of ENOD40 s leads to significant suppression of nodule formation in Lotus japonicus. *Plant Cell Physiol.* 47 1102–1111. 10.1093/pcp/pcj081 16816411

[B18] Lefebvre B. Timmers T. Mbengue M. Moreau S. Hervé C. Tóth K. (2010). A remorin protein interacts with symbiotic receptors and regulates bacterial infection. *Proc. Natl. Acad. Sci. U.S.A.* 107 2343–2348. 10.1073/pnas.0913320107 20133878 PMC2836688

[B19] Lerouge P. Roche P. Faucher C. Maillet F. Truchet G. Promé J. C. (1990). Symbiotic host-specificity of Rhizobium meliloti is determined by a sulphated and acylated glucosamine oligosaccharide signal. *Nature* 344 781–784. 10.1038/344781a0 2330031

[B20] Li Z. Cao R. Wang M. Myers M. P. Zhang Y. Xu R.-M. (2006). Structure of a Bmi-1-Ring1B polycomb group ubiquitin ligase complex. *J. Biol. Chem.* 281 20643–20649. 10.1074/jbc.M602461200 16714294

[B21] Liew R. Chu W. Sun H. Hunter T. Catherine L. (2010). RING domain dimerization is essential for RNF4 function. *Biochem. J.* 431 23–29. 10.1042/bj20100957 20681948 PMC3104014

[B22] Long S. R. (1989). Rhizobium-legume nodulation: life together in the underground. *Cell* 56 203–214. 10.1016/0092-8674(89)90893-3 2643474

[B23] Mace P. D. Linke K. Feltham R. Schumacher F.-R. Smith C. A. Vaux D. L. (2008). Structures of the cIAP2 RING domain reveal conformational changes associated with ubiquitin-conjugating enzyme (E2) recruitment. *J. Biol. Chem.* 283 31633–31640. 10.1074/jbc.M804753200 18784070

[B24] Masson-Boivin C. Giraud E. Perret X. Batut J. (2009). Establishing nitrogen-fixing symbiosis with legumes: how many rhizobium recipes? *Trends Microbiol.* 17 458–466. 10.1016/j.tim.2009.07.004 19766492

[B25] Mbengue M. Camut S. de Carvalho-Niebel F. Deslandes L. Froidure S. Klaus-Heisen D. (2010). The Medicago truncatula E3 ubiquitin ligase PUB1 interacts with the LYK3 symbiotic receptor and negatively regulates infection and nodulation. *Plant Cell* 22 3474–3488. 10.1105/tpc.110.075861 20971894 PMC2990133

[B26] McGinnes L. W. Morrison T. G. (1998). Role of carbohydrate processing and calnexin binding in the folding and activity of the HN protein of new castle disease virus. *Virus Res.* 53 175–185. 10.1016/S0168-1702(97)00144-5 9620209

[B27] Oldroyd G. E. Downie J. A. (2004). Calcium, kinases and nodulation signalling in legumes. *Nat. Rev. Mol. Cell Biol.* 5 566–576. 10.1038/nrm1424 15232574

[B28] Oldroyd G. E. Downie J. A. (2006). Nuclear calcium changes at the core of symbiosis signalling. *Curr. Opin. Plant Biol.* 9 351–357. 16713329 10.1016/j.pbi.2006.05.003

[B29] Oldroyd G. E. Downie J. A. (2008). Coordinating nodule morphogenesis with rhizobial infection in legumes. *Annu. Rev. Plant Biol.* 59 519–546. 10.1146/annurev.arplant.59.032607.092839 18444906

[B30] Petroski M. D. Deshaies R. J. (2005). Function and regulation of cullin–RING ubiquitin ligases. *Nat. Rev. Mol. Cell Biol.* 6 9–20. 10.1038/nrm1547 15688063

[B31] Plechanovová A. Jaffray E. G. McMahon S. A. Johnson K. A. Navrátilová I. Naismith J. H. (2011). Mechanism of ubiquitylation by dimeric RING ligase RNF4. *Nat. Struct. Mol. Biol.* 18 1052–1059. 10.1038/nsmb.2108 21857666 PMC3326525

[B32] Radutoiu S. Madsen L. H. Madsen E. B. Felle H. H. Umehara Y. Gronlund M. (2003). Plant recognition of symbiotic bacteria requires two LysM receptor-like kinases. *Nature* 425 585–592. 10.1038/nature02039 14534578

[B33] Schwechheimer C. (2008). Understanding gibberellic acid signaling-are we there yet? *Curr. Opin. Plant Biol.* 11 9–15. 10.1016/j.pbi.2007.10.011 18077204

[B34] Shimomura K. Nomura M. Tajima S. Kouchi H. (2006). LjnsRING, a novel RING finger protein, is required for symbiotic interactions between Mesorhizobium loti and Lotus japonicus. *Plant Cell Physiol.* 47 1572–1581. 17056617 10.1093/pcp/pcl022

[B35] Stracke S. Kistner C. Yoshida S. Mulder L. Sato S. Kaneko T. (2002). A plant receptor-like kinase required for both bacterial and fungal symbiosis. *Nature* 417 959–962. 12087405 10.1038/nature00841

[B36] Tansengco M. L. Hayashi M. Kawaguchi M. Imaizumi-Anraku H. Murooka Y. (2003). crinkle, a novel symbiotic mutant that affects the infection thread growth and alters the root hair, trichome, and seed development in Lotus japonicus. *Plant Physiol.* 131 1054–1063. 10.1104/pp.102.017020 12644658 PMC166871

[B37] Tsikou D. Ramirez E. E. Psarrakou I. S. Wong J. E. Jensen D. B. Isono E. (2018). A Lotus japonicus E3 ligase interacts with the Nod Factor Receptor 5 and positively regulates nodulation. *BMC Plant Biol.* 18:217. 10.1186/s12870-018-1425-z 30285618 PMC6171183

[B38] Vander Kooi C. W. Ohi M. D. Rosenberg J. A. Oldham M. L. Newcomer M. E. Gould K. L. (2006). The Prp19 U-box crystal structure suggests a common dimeric architecture for a class of oligomeric E3 ubiquitin ligases. *Biochemistry* 45 121–130. 10.1021/bi051787e 16388587 PMC2570371

[B39] Vernié T. Camut S. Camps C. Rembliere C. de Carvalho-Niebel F. Mbengue M. (2016). PUB1 interacts with the receptor kinase DMI2 and negatively regulates rhizobial and arbuscular mycorrhizal symbioses through its ubiquitination activity in *Medicago truncatula*. *Plant Physiol.* 170 2312–2324. 10.1104/pp.15.01694 26839127 PMC4825150

[B40] Vinardell J. M. Fedorova E. Cebolla A. Kevei Z. Horvath G. Kelemen Z. (2003). Endoreduplication mediated by the anaphase-promoting complex activator CCS52A is required for symbiotic cell differentiation in *Medicago truncatula* nodules. *Plant Cell* 15 2093–2105. 10.1105/tpc.014373 12953113 PMC181333

[B41] Waadt R. Schmidt L. K. Lohse M. Hashimoto K. Bock R. Kudla J. (2008). Multicolor bimolecular fluorescence complementation reveals simultaneous formation of alternative CBL/CIPK complexes in planta. *Plant J.* 56 505–516. 10.1111/j.1365-313X.2008.03612.x 18643980

[B42] Wang C. Zhu H. Jin L. Chen T. Wang L. Kang H. (2013). Splice variants of the SIP1 transcripts play a role in nodule organogenesis in *Lotus japonicus*. *Plant Mol. Biol.* 82 97–111. 10.1007/s11103-013-0042-3 23494209

[B43] Xie Q. Guo H.-S. Dallman G. Fang S. Weissman A. M. Chua N.-H. (2002). SINAT5 promotes ubiquitin-related degradation of NAC1 to attenuate auxin signals. *Nature* 419 167–170. 10.1038/nature00998 12226665

[B44] Yano K. Shibata S. Chen W. L. Sato S. Kaneko T. Jurkiewicz A. (2009). CERBERUS, a novel U-box protein containing WD-40 repeats, is required for formation of the infection thread and nodule development in the legume-Rhizobium symbiosis. *Plant J.* 60 168–180. 19508425 10.1111/j.1365-313X.2009.03943.x

[B45] Yin Q. Lin S.-C. Lamothe B. Lu M. Lo Y.-C. Hura G. (2009). E2 interaction and dimerization in the crystal structure of TRAF6. *Nat. Struct. Mol. Biol.* 16 658–666. 10.1038/nsmb.1605 19465916 PMC2834951

[B46] Yoshida S. Parniske M. (2005). Regulation of plant symbiosis receptor kinase through serine and threonine phosphorylation. *J. Biol. Chem.* 280 9203–9209. 10.1074/jbc.M411665200 15572355

[B47] Yuan S. Zhu H. Gou H. Fu W. Liu L. Chen T. (2012). A ubiquitin ligase of symbiosis receptor kinase involved in nodule organogenesis. *Plant Physiol.* 160 106–117. 10.1104/pp.112.199000 22822209 PMC3440188

[B48] Zheng N. (2003). A closer look of the HECTic ubiquitin ligases. *Structure* 11 5–6. 10.1016/s0969-2126(02)00940-1 12517333

[B49] Zhu H. Chen T. Zhu M. Fang Q. Kang H. Hong Z. (2008). A novel ARID DNA-binding protein interacts with SymRK and is expressed during early nodule development in *Lotus japonicus*. *Plant Physiol.* 148 337–347. 10.1104/pp.108.119164 18633121 PMC2528112

