# Supplementary figures and images for: The Lotus japonicus Ubiquitin Ligase SIE3 Interacts With the Transcription Factor SIP1 and Forms a Homodimer
Source: Front Plant Sci. 2020 Jun 12;11:795. doi: 10.3389/fpls.2020.00795 (PMC7303358; doi:10.3389/fpls.2020.00795)

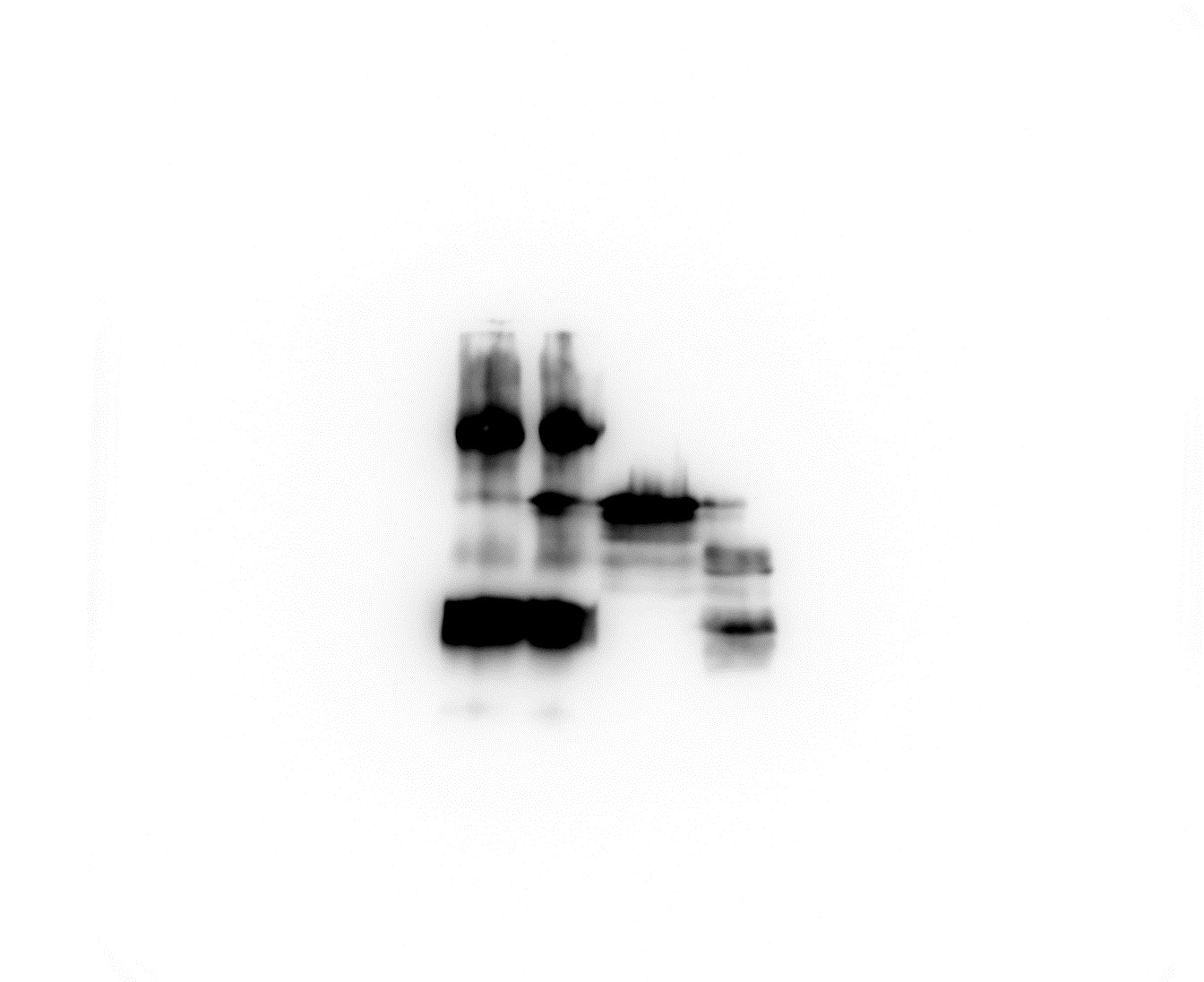

Supplement: Supplementary file 1 [file Data_Sheet_1.ZIP › Figure 1B a-HA(AD).jpg]

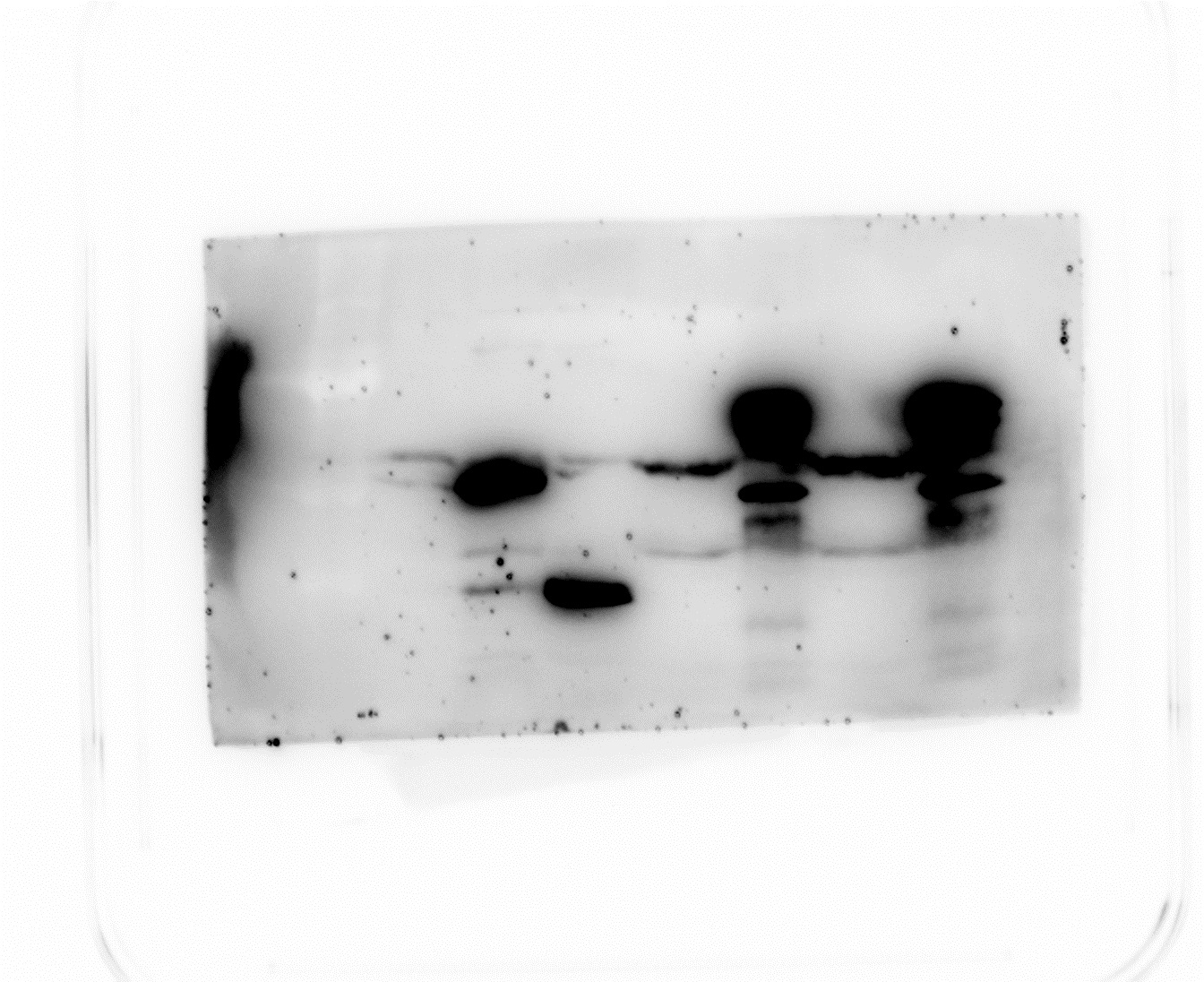

Supplement: Supplementary file 1 [file Data_Sheet_1.ZIP › Figure 1B a-Myc (BD).jpg]

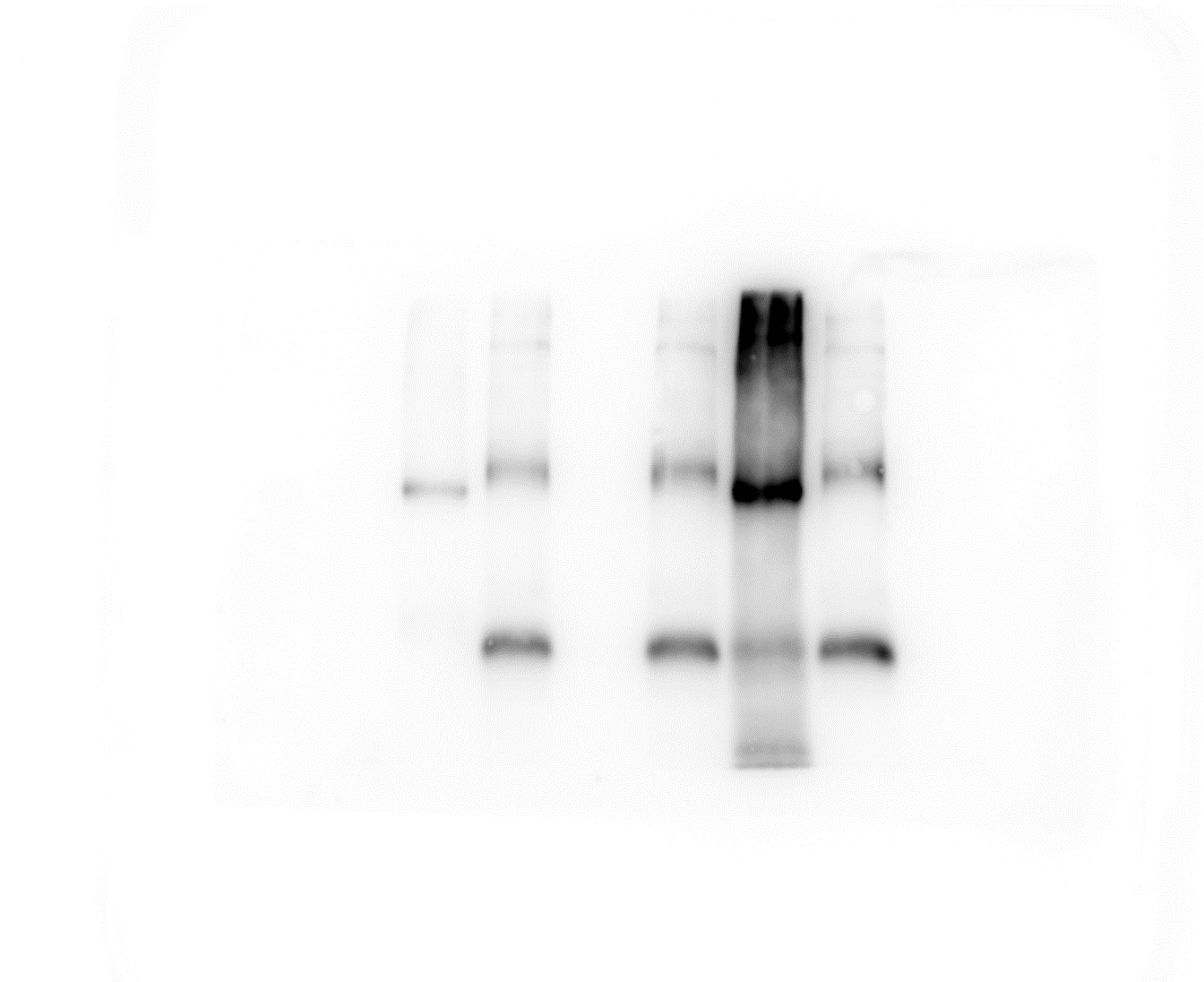

Supplement: Supplementary file 1 [file Data_Sheet_1.ZIP › Figure 2B a-FLAG (SCN).jpg]

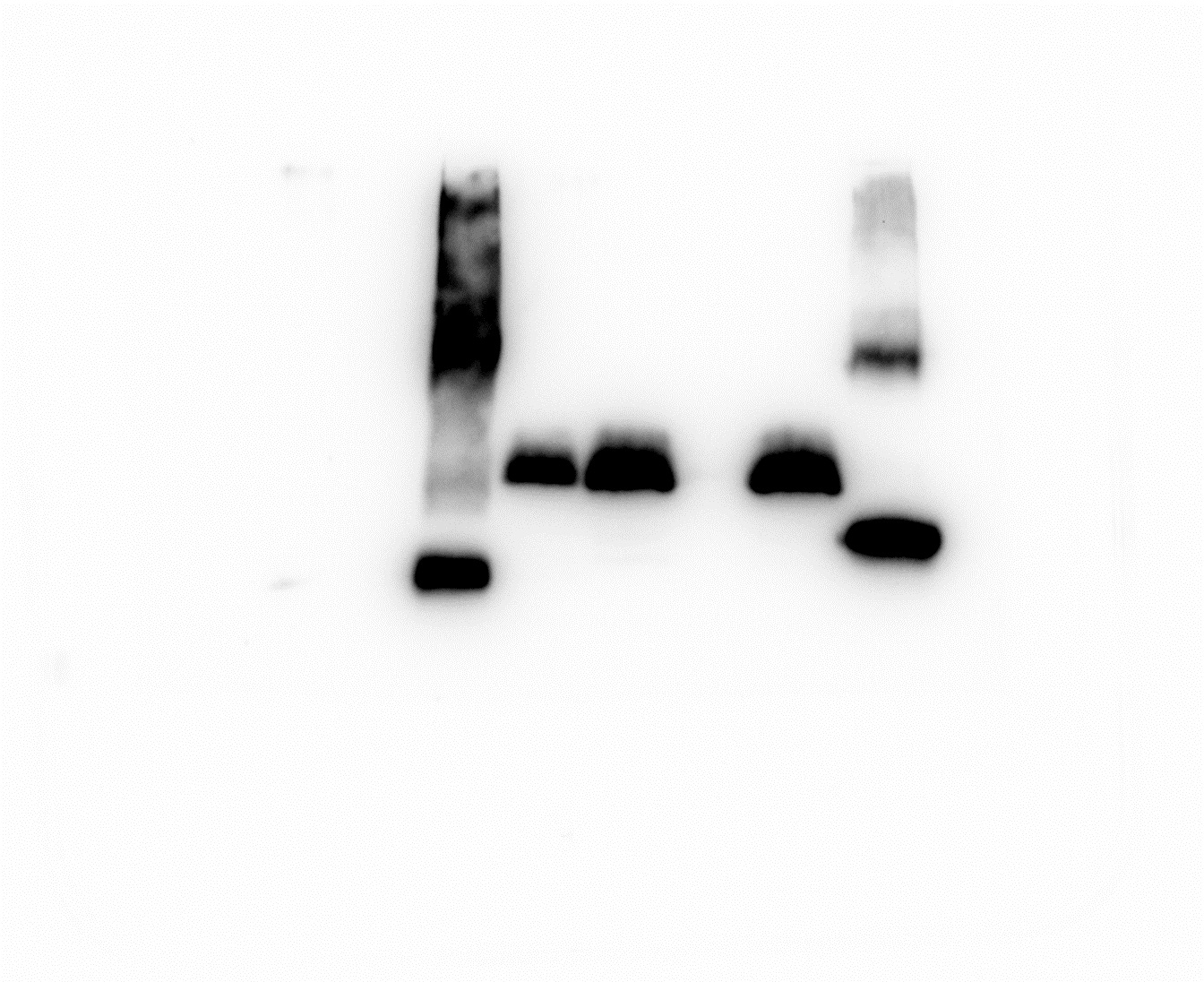

Supplement: Supplementary file 1 [file Data_Sheet_1.ZIP › Figure 2B a-HA (SCC).jpg]

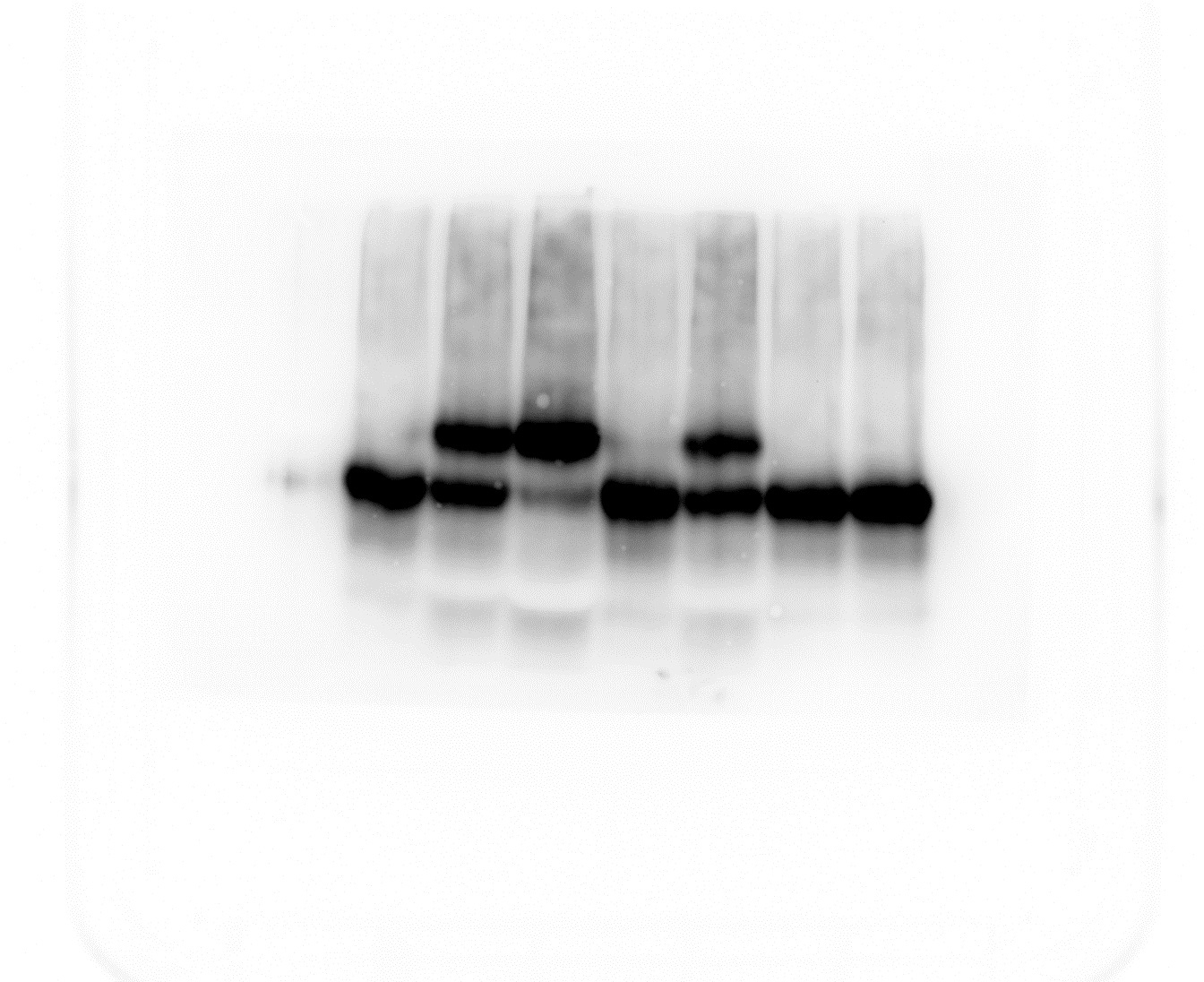

Supplement: Supplementary file 1 [file Data_Sheet_1.ZIP › Figure 3 a-GFP.jpg]

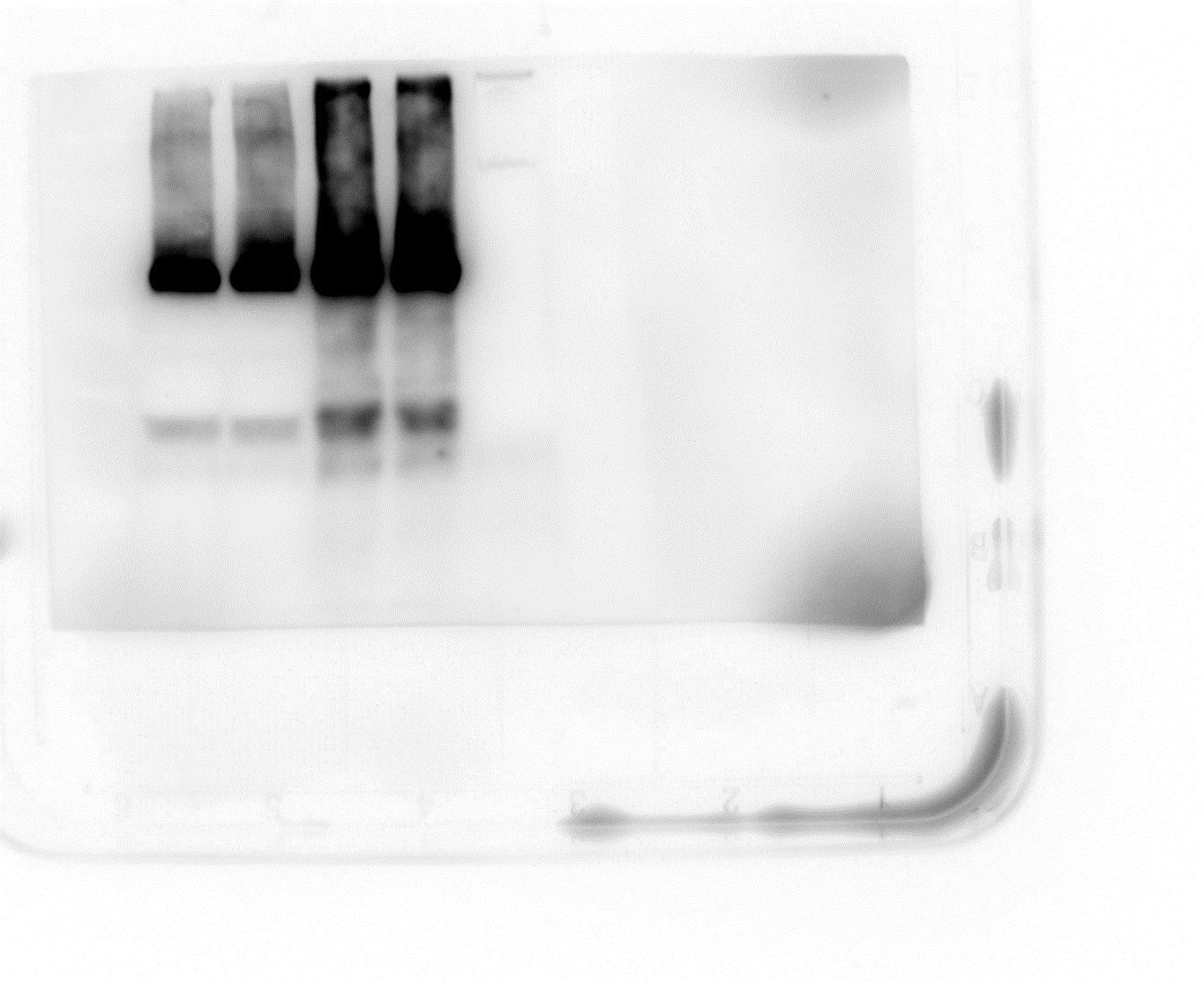

Supplement: Supplementary file 1 [file Data_Sheet_1.ZIP › Figure 5B a-FLAG (SCC).jpg]

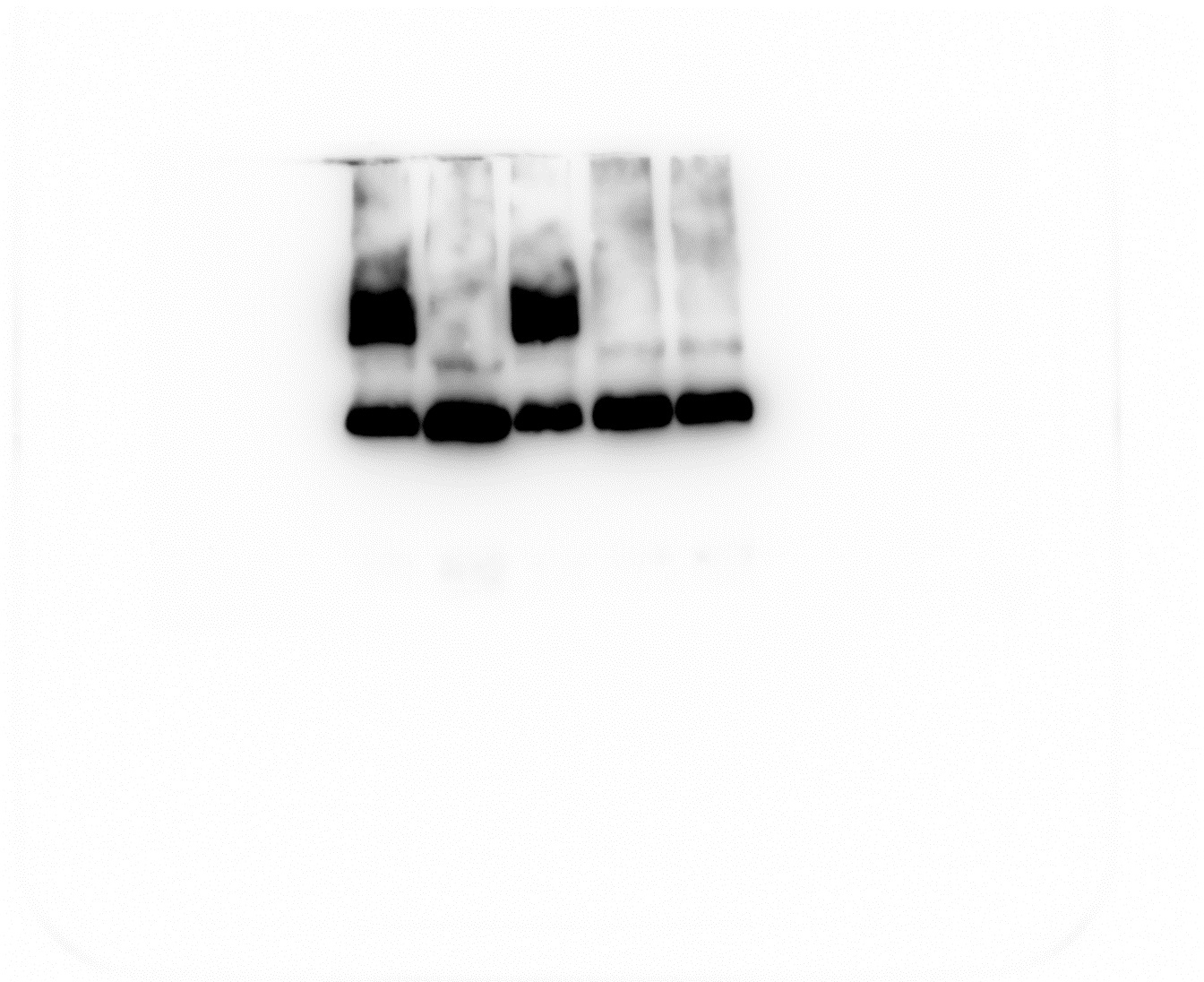

Supplement: Supplementary file 1 [file Data_Sheet_1.ZIP › Figure 5B a-HA (SCN).jpg]
